# An underutilized orphan tuber crop—Chinese yam : a review

**DOI:** 10.1007/s00425-020-03458-3

**Published:** 2020-09-21

**Authors:** Janina Epping, Natalie Laibach

**Affiliations:** 1 Institute of Plant Biology and Biotechnology, University of Muenster, Schlossplatz 8, 48143 Muenster, Germany; 2 Institute for Food and Resource Economics, University of Bonn, Meckenheimer Allee 174, 53115 Bonn, Germany

**Keywords:** *Dioscorea polystachya*, Chinese yam, Functional food, Orphan crop

## Abstract

**Main conclusion:**

The diversification of food crops can improve our diets and address the effects of climate change, and in this context the orphan crop Chinese yam shows significant potential as a functional food.

**Abstract:**

As the effects of climate change become increasingly visible even in temperate regions, there is an urgent need to diversify our crops in order to address hunger and malnutrition. This has led to the re-evaluation of neglected species such as Chinese yam (*Dioscorea polystachya* Turcz.), which has been cultivated for centuries in East Asia as a food crop and as a widely-used ingredient in traditional Chinese medicine. The tubers are rich in nutrients, but also contain bioactive metabolites such as resistant starches, steroidal sapogenins (like diosgenin), the storage protein dioscorin, and mucilage polysaccharides. These health-promoting products can help to prevent cardiovascular disease, diabetes, and disorders of the gut microbiome. Whereas most edible yams are tropical species, Chinese yam could be cultivated widely in Europe and other temperate regions to take advantage of its nutritional and bioactive properties. However, this is a laborious process and agronomic knowledge is fragmented. The underground tubers contain most of the starch, but are vulnerable to breaking and thus difficult to harvest. Breeding to improve tuber shape is complex given the dioecious nature of the species, the mostly vegetative reproduction via bulbils, and the presence of more than 100 chromosomes. Protocols have yet to be established for in vitro cultivation and genetic transformation, which limits the scope of research. This article summarizes the sparse research landscape and evaluates the nutritional and medical applications of Chinese yam. By highlighting the potential of Chinese yam tubers, we aim to encourage the adoption of this orphan crop as a novel functional food.

## Introduction

Grains and vegetables provide a rich source of calories and nutrients, but many people subsist on monotonous cereal diets and are therefore nutritionally deprived even if they ingest sufficient calories. Increasing the diversity of crops on global and local markets is one of the major challenges for agriculture, especially in the face of climate change (Hunter et al. 2019; Mabhaudhi et al. 2019). So-called orphan crops are underutilized species that have local significance, especially for small-scale farmers, but are neglected on a global scale (Mabhaudhi et al. 2019). They are often overlooked by researchers, despite valuable traits that are promising for emerging markets (Tadele 2019). Orphan crops offer greater nutritional value that could improve and diversify our diets (Hunter et al. 2019). Some of them also have potential as functional foods and could capture new markets (Aditya et al. 2019). One such orphan crop is the Chinese yam, *Dioscorea polystachya* Turcz. The tubers of this crop are highly nutritious but they also contain health-promoting secondary metabolites that provide additional health benefits such as the regulation of blood sugar levels and the control of cholesterol, fat uptake and hypertension (Chen et al. 2003; Shujun et al. 2008; Amat et al. 2014; Zhang et al. 2016). These properties reflect the high content of resistant starches, polysaccharides, and steroidal sapogenins, which could allow the development of Chinese yam as a functional food for the prevention of diabetes and heart disease. The tubers can be prepared in the same way as potatoes, or can be used as a food additive. Chinese yam is a well-known vegetable in East Asia, especially in China where it has been used as food and in traditional medicine for thousands of years (Amat et al. 2014), but it is almost unknown to the rest of the world. Several yam species have become staple foods in West Africa, but Chinese yam is the only edible yam species that can be cultivated in temperate regions such as Europe. However, the cultivation of this crop is challenging, which has hampered its commercial development beyond East Asia. In this review, we highlight the unique properties of Chinese yam tubers, as well as discussing the genetics, breeding options and cultivation of this orphan crop. We also evaluate the potential of Chinese yam as a functional food for global markets, based on its cultivation in temperate regions.

## Global relevance and cultivation of yam species

Yams (*Dioscorea* spp.) are distributed almost ubiquitously in the tropics and subtropics but few species can grow in temperate regions (Wilkin et al. 2005). Yams are cultivated as food crops in Africa, Asia, parts of South America and the Caribbean, and in the South Pacific islands (Asiedu and Sartie 2010). However, yam cultivation in other places may not been reported, so these statistics may not provide the full picture of the geographical scope of yam cultivation. Production is concentrated in Nigeria, Ghana, Benin, the Republic of Côte d’Ivore, Cameroon and Togo, which together comprise the so called ‘yam belt’ (Scarcelli et al. 2019). In these countries, yams are not only staple foods but also have important cultural and social functions (Obidiegwu and Akpabio 2017).

Nigeria tops the list of producers, with almost 48 million tonnes harvested in 2018 (FAOSTAT). Global production has increased dramatically over the last 20 years, starting from ~ 17 million tonnes in 1988 to reach 73 million tonnes in 2018 (FAOSTAT). This partially reflects the efforts of organizations such as the International Institute of Tropical Agriculture (IITA), which focuses on maximizing the profitability of yam production (Mignouna et al. 2009). Despite its growing importance, research on yam crops has been neglected for a long time and modern breeding programs based on genomics have been applied to yams only in the last few years (Darkwa et al. 2019).

Chinese yam is unusual in that it is indigenous to temperate regions and tolerates much colder temperatures than its relatives. The species is native to China, Korea, the Kuril Islands and Taiwan, but has been introduced into Japan, the south-east USA, Uruguay and Western Himalaya (Coursey 1967a; POWO 2019). In China, it is called ‘Huai Shan Yao’ (Zhang et al. 2011). In Japan, Chinese yam cultivars are distinguished by tuber shape, namely Nagaimo (cylindrical), Tsukuneimo (round) and Ichoimo (flattened) (Babil et al. 2013). Here we use the binomial designation *Dioscorea polystachya* Turcz., but various synonyms are used in the literature, including *D. batatas*, *D. pseudobatatas*, *D. rosthornii* and *D. swinhoei*, which often leads to confusion and disagreement (Ting and Gilbert 2000; Hsu et al. 2013). In East Asian literature, the species is usually named *D. opposita* Thunb., but according to the ‘world checklist of selected plant families’ this is now regarded as a synonym for *D. oppositifolia*, a yam species native to India (Kew Science; Ting and Gilbert 2000). Furthermore, *D. esuclenta* and *D. bulbifera* are known as ‘Chinese yam’ in some regions, whereas the name ‘cinnamon vine’ typically refers to *D. polystachya* Turcz. but is also used for other yam species in the Americas, increasing the degree of confusion (Coursey 1967a). For clarity, we use Chinese yam to mean *Dioscorea polystachya* Turcz. throughout this article. The closely related Japanese yam (*D. japonica* Thunb.) is native to South China, Japan, Korea and Taiwan (Kew Science; Hsu et al. 2013). In Japan it is called ‘yamanoimo’ (Kawasaki et al. 2001). This species can also grow in temperate climates and is widely cultivated as a food crop, and the review also covers some studies that focus on this species.

Several cultivars of Chinese yam are grown for food and medical purposes (Kawasaki et al. 2014). The Henan province in China is the main production area and is associated with the highest quality products (Zhang et al. 2014). In China, the cultivar ‘Tiegun’ is most popular because it has superior nutritional and pharmaceutical properties and has been used to treat conditions such as diarrhea, diabetes and asthma for more than 2000 years (Peng et al. 2017). In China, producers are currently unable to meet the high demand for this valuable product (Zhang et al. 2014). Depending on the cultivar, Chinese yam tubers comprise ~ 65% starch, ~ 9% protein and ~ 1.2% fiber, and also provide a rich source of minerals (e.g., ~ 470 mg/kg Ca and Mg) and bioactive compounds such as allantoin and dioscin, which account for 0.8% and 0.077% of the dry weight, respectively (Wu et al. 2016). In contrast to other edible yams, Chinese yam tubers are non-toxic and can be eaten raw (Chan and Ng 2013). The peel is usually removed, and the remaining flesh has a sticky texture reflecting the high quantity of mucilage, which is appreciated in East Asia because it lends texture to soup.

## Botanical and physiological properties

**Fig. 1:**
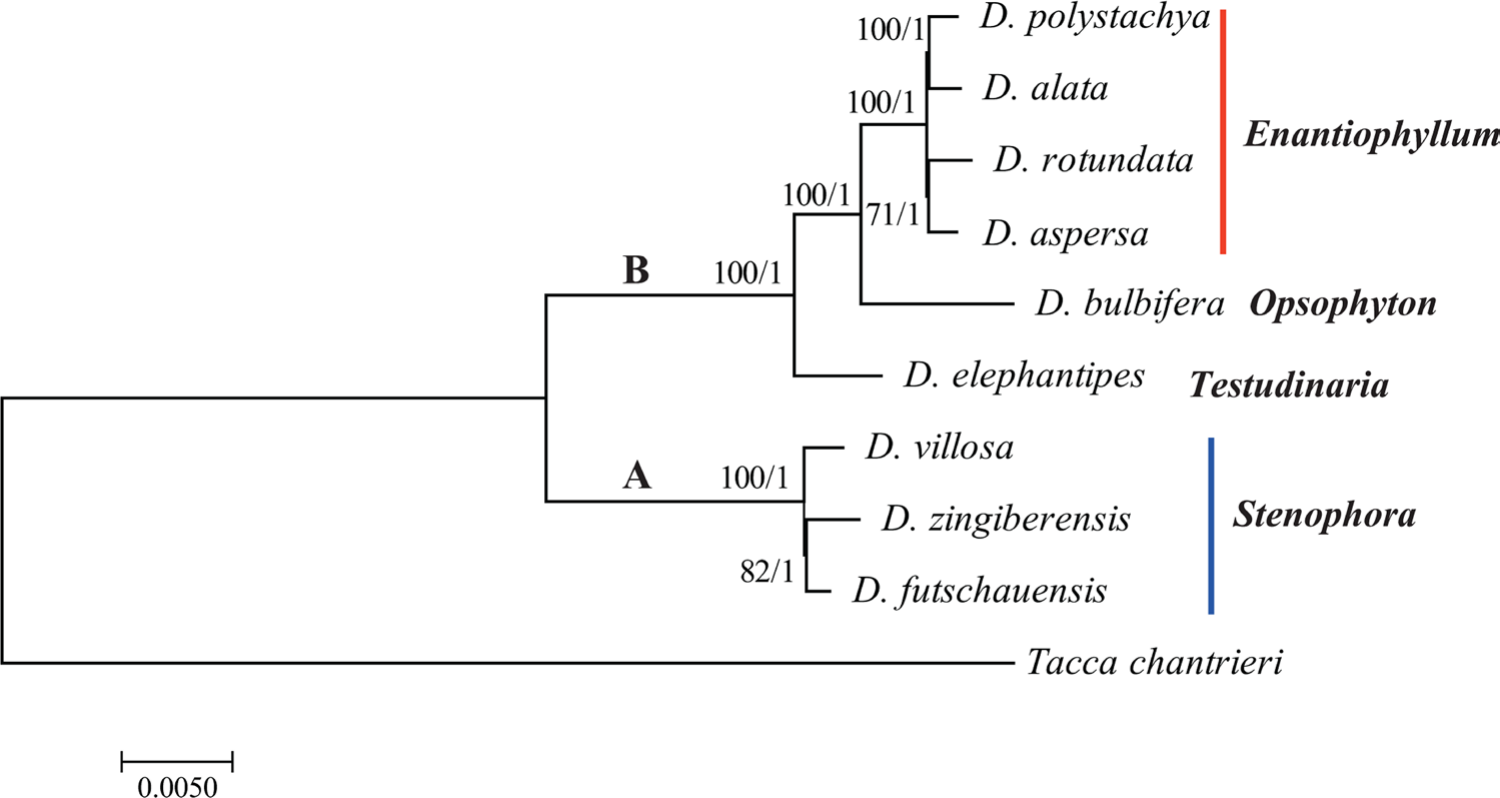
Phylogenetic tree based on the complete chloroplast genome sequences of 9 *Dioscorea* species. Maximum likelihood (ML) topology shown with ML bootstrap support values/Bayesian posterior probability listed at each node. *Dioscorea* species were grouped into clade A and B. Figure taken from (Zhao et al. 2018)

The family Dioscoreaceae includes > 650 species in 4 genera, including the large yam genus *Dioscorea* with about 625 species (Caddick et al. 2002; Wilkin et al. 2005; POWO 2019). Within this genus the Chinese yam belongs to the section Enantiophyllum, which includes two important food staples: the water yam *D. alata* and the white guinea yam *D. rotundata* (Coursey 1967a; Wilkin et al. 2005; Govaerts et al. 2007; Viruel et al. 2016). Phylogenetic analysis using complete chloroplast genome sequences show that these three species are closely related within the section Enantiophyllum (Fig. 1). Yams are monocotyledonous twining vines that use other plants for support, and their stems can climb to a height of > 3 m (Coursey 1967a, b; Mueller et al. 2003). Some yam species form starchy underground tubers and sometimes aerial tubers (bulbils) in their leaf axils, which are used for propagation (Ding and Gilbert 2000). This has facilitated their domestication for food and medical use (Coursey 1967a). They grow annually and the tubers enter a long phase of dormancy at the end of the season before they re-sprout. This prolonged dormancy allows wild yams to be used as a famine food in West Africa (Martin and Degras 1978). Most yam plants are dioecious, meaning that male pistil-bearing flowers and female stamina-bearing flowers form on separate plants, although occasionally monoecious and non-flowering cultivars can be found (Coursey 1967a). Yam flowers are small (2–4 mm in diameter) and are thought to be naturally pollinated by thrips and other small insects (Coursey 1967a; Segnou et al. 1992; Mizuki et al. 2005a). Chinese yam plants flower between June and September, with fruit set between July and November (Ting and Gilbert 2000). Yam fruits are trilocular capsules, 1–3 cm in length, that contain small, light and flattened seeds (Coursey 1967a). True seeds are produced when male and female plants grow in close proximity, but clonal reproduction and vegetative propagation are far more common (Mizuki et al. 2010; Walck et al. 2010). In its natural habitat, the Chinese yam grows in forests, on mountain slopes, and along rivers and roadsides (Ting and Gilbert 2000).

Yam genetics is poorly understood because most yam species are polyploid with a long growth cycle, infrequent flowering, a heterozygous genetic background, and predominant vegetative propagation (Mignouna et al. 2003). Species of the genus *Dioscorea* show high variation of ploidy levels among and within species (Gamiette et al. 1999; Dansi et al. 2001; Obidiegwu et al. 2009; Viruel et al. 2019). The most common food yams have ploidy levels of 4 × and 6 × for *D. alata* (Viruel et al. 2019), 4×, 6 × and 8 × for *D. cayenensis*/*D. rotundata* complex (Dansi et al. 2001; Obidiegwu et al. 2009; Viruel et al. 2019) and 2 × and 8 × for *D. bulbifera* (Viruel et al. 2019). Reported base numbers include *x* = 6, 7, 9, 10 and 20 (*x* = number of chromosomes per single monoploid genome) with *x* = 10 being the most common base number among cultivated species (Martin and Ortiz 1963; Viruel et al. 2008; Arnau et al. 2009). For Chinese yam and *D. japonica* a base number of *x* = 10 with chromosome numbers of 2n = 100 or 140 for Chinese yam (Babil et al. 2013; Viruel et al. 2019) and 2n = 40, 80 and 100 for *D. japonica* have been reported (Nakajima 1934; Araki et al. 1983).

### Tuber anatomy and growth

Yam tubers have unique morphological and physiological characteristics (Onwueme 1979). Whereas the storage organs of most root and tuber crops originate from the roots (sweet potato and cassava), stem (potato) or corm (taro), yam tubers are derived from the hypocotyl (Lawton and Lawton 1969; Shewry 2003). The tubers of Chinese yam feature a thin brown skin made of cork cells that covers the inner white flesh (Raman et al. 2014). Small roots on the tuber surface confer a hairy appearance (Coursey 1967a). The skin comprises several cork layers and a band of lignified sclerenchymal cells. The cork cambium features two layers of tangentially elongated parenchymal cells and the cortex consists of compact cells arranged in 6–8 layers. The ground tissue is wide and filled with starch grains, with a small number of irregularly distributed vascular bundles. Many idioblasts containing calcium oxalate embedded in mucilage are distrusted in the cortex and ground tissue. The starch grains are oval, elliptic or shell-shaped, 10–39 µm long and 7–29 µm wide (Raman et al. 2014).

The tubers of Chinese yam are usually spindle-shaped or club-like, and may grow up to a meter in length, but they are comparatively thin (ca. 5–8 cm (Araki et al. 1983)) and grow almost vertically (in the direction of gravity), resembling the growth of a primary root (Coursey 1967a; Kawasaki et al. 2008). Harvesting is therefore labor-intensive because the tubers must be dug out of the ground while avoiding damage. In Japan, the tubers are often cultivated in horizontal pipes that protect them, guide their growth, and facilitate harvesting (Kadota and Niimi 2004). However, the tuber shape is cultivar-dependent, in some cases thin and elongated, and in others thicker, flattened or even oval, with the latter being easier to harvest (Kawasaki et al. 2008; Babil et al. 2013). The tuber shape depends on factors such as the density, porosity and chemical properties of the soil, and the presence of obstacles in the soil (which lead to deformation). Chinese yam tubers grow only at the tip, which contains apical meristem that proliferates continually while the upper tuber part enters dormancy, creating a tuber with a thin top and a thick base. The tuber tip exhibits positive gravitropism, and amyloplasts in the tip undergo sedimentation along the gravity vector, suggesting they serve as gravity-sensing statoliths that guide tuber formation (Kawasaki et al. 2008). Additional gravistimulation experiments revealed that only the subapical part of the tubers shows a response to gravity. This is similar to the distal elongation zone near root caps, where the curvature response occurs after gravistimulation (Kawasaki et al. 2014). The gravisensing site in the tuber tip may change during tuber growth in some cultivars. For example, tuber growth in the cultivar Genkotsujirou is thought to switch between elongation and thickening phases, whereas most cultivars remain in the elongation phase. In cultivars that feature a thickening phase, the tuber tip possesses fewer amyloplasts indicating a loss of gravisensing ability (Kawasaki et al. 2008). Gravistimulation experiments in which tubers were reoriented showed that the tuber tip finds its way back to the gravity vector by changing the direction of growth. This was accompanied by changes in amyloplast localization, supporting their role in gravisensing and tuber growth. Interestingly, changing the tuber orientation in short intervals caused the tuber to grow several tips that extended in different directions (Kawasaki et al. 2014). To suppress shoot production by gravitropic disorientation, this may be connected to upside-down or vertical storage practices of yam tubers (Coursey 1967b).

### Life cycle and cultivation

**Fig. 2:**
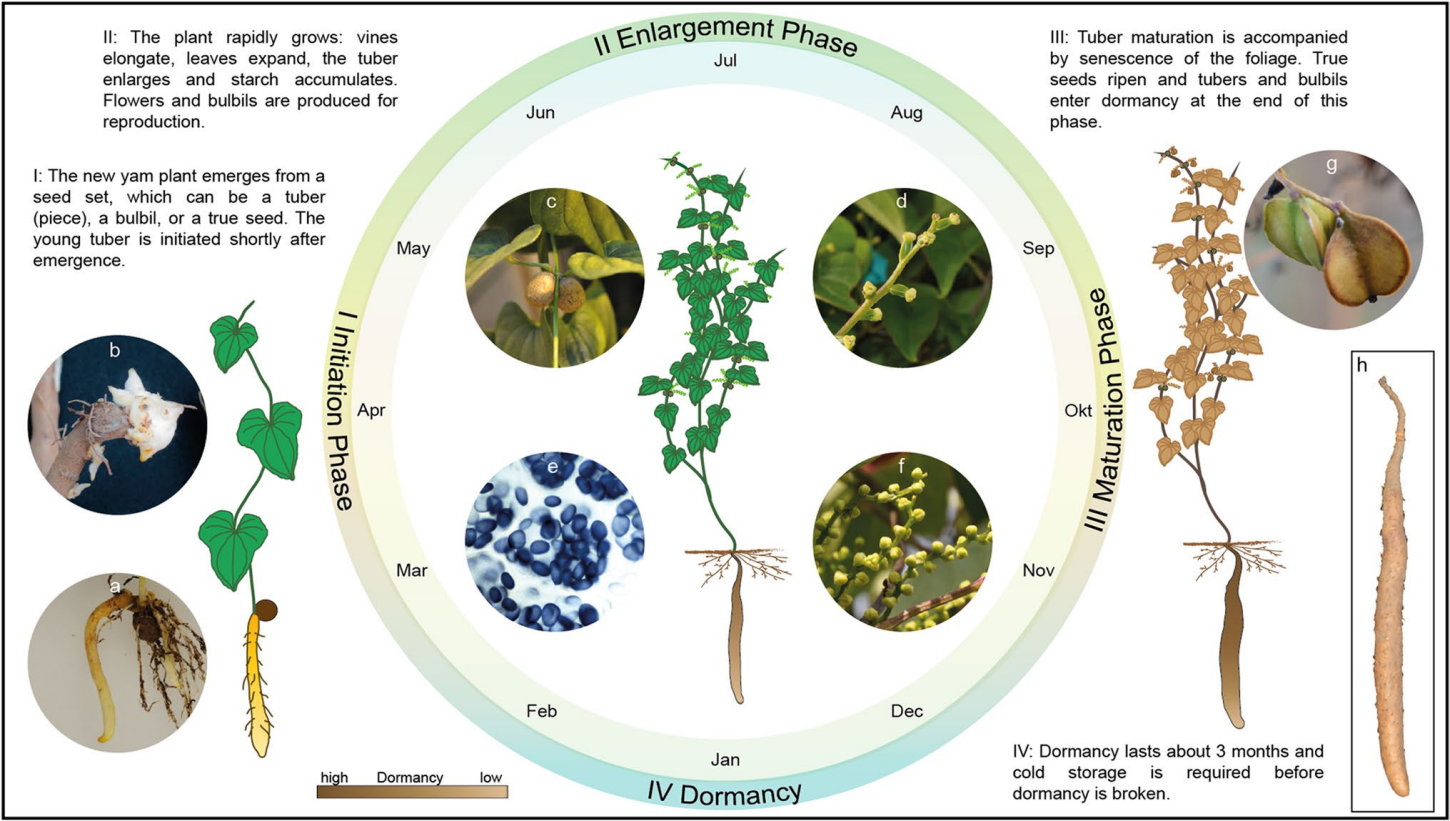
Growth cycle of Chinese yam. **a**: Young tuber 25 days after emergence. **b**: Bud at a tuber head. **c**: Bulbils in leaf axils. **d**: Female inflorescence. **e**: Starch granules in the tuber stele. **f**: Male inflorescence. **g**: Seed capsules. **h**: Mature tuber

Yams that are used as field crops are basically perennial plant, but are often cultivated annually (Coursey 1967a). Tubers and bulbils can be used as propagation organs (Kim et al. 2003a). If Chinese yam plants are grown from bulbils, the resulting tubers are small but can be used as set seed in the 2nd year. When large tuber pieces or whole tubers are used for seed setting, the new tubers can exceed 1 m in length and weigh more than 3 kg. In the northern hemisphere, Chinese yam sets are usually planted in spring and grow over the summer months until they start to wilt in autumn. Tubers and bulbils are dormant at maturity, and cold stratification is required for germination in the following spring (Okagami and Tanno 1991). The growth of tropical and temperate edible yams is in principle similar and occurs in four phases (Asiedu et al. 1998; Gong et al. 2017), which are shown for Chinese yam in **Fig. ****2**.

#### Phase I: initiation (March–April)

The life cycle starts when the new plant emerges from the post-dormant tuber. The physiological processes involved in tuber sprouting have been well described in several *Dioscorea* species (Lawton and Lawton 1969). In contrast to potato, yam tubers do not produce dormant buds. Instead, sprouting involves the activity of a layer of meristematic cells located just beneath the tuber skin (Onwueme 1973; Wickham et al. 1981). One or a few buds are formed de novo just before sprouting, usually in the head region first because sprouting yam tubers exhibit proximal dominance (Onwueme 1979). This is caused by a gradient in the level of dormancy: the tuber tip proliferates continually but the head region enters dormancy, thus the dormancy period starts earlier and there is less time between harvest and sprouting. When the first sprouts form, the formation of subsequent sprouts is repressed. The tuber heads are therefore preferred as seed tubers because they will sprout earlier than the middle and tail regions. This is important for yam multiplication because cutting the tuber into segments just before planting will lead to heterogeneous and delayed sprouting, reflecting the need for the middle or tail parts to differentiate into new buds. For African yam, multiplication techniques such as minisetting with small tuber pieces have been developed to overcome the otherwise low multiplication rates (Onwueme 1973,1979; Aighewi et al. 2015).

The sprout becomes visible as a swollen structure known as the primary nodal complex, from which the yam vines, feeder roots, and the new tuber emerge (Hamadina 2012). Tuber initiation is characterized by rapid proliferation of the meristematic tissue at the junction between the stem and root, which soon forms an organized structure with a set proximal-to-distal (head-to-tail) polarity. The distal end is geotropic and grows downwards, whereas the proximal end hardens (Asiedu et al. 1998). The timing of tuber initiation depends on the species and environmental factors (Asiedu et al. 1998). In Chinese yam, tuber initiation begins as early as 20 days after the field planting of tuber pieces with buds (Gong et al. 2017). Upon further growth, the old tuber shrivels as it supplies the new plant with nutrients (Asiedu et al. 1998).

#### Phase II: enlargement (May–September)

Phase II is characterized by rapid cell division and expansion. The vine elongates, leaves expand, and tubers enlarge (Asiedu et al. 1998). Later in this phase, bulbils and flowers can be produced. The development of bulbils in Chinese yam plants is dependent on the cultivar, but the ability to flower is dependent on the size of the seed tuber. Depending on the cultivar, the minimum weight for floral initiation is 40–75 g (Yoshida et al. 1996). This is relevant for breeding because plants that grow from bulbils or true seeds tend not to flower in the first year.

During the enlargement phase, Chinese yam tubers grow slowly for 40–60 days, then enter a rapid growth period lasting ~ 60 days before leveling off and reaching a plateau over the next ~ 30 days (Onwueme 1979). During the early stages of the rapid growth period, the tuber increases significantly in length and to a lesser extent in width and weight (Gong et al. 2017). At the end of this phase, the maximum canopy is reached and the tubers rapidly accumulate dry matter. Here, the maximum tuber yield is determined by the rate of photosynthesis and translocation of assimilates from source tissues (Asiedu et al. 1998). The tuber does not accumulate starch during the initiation phase but starch grains are formed in the parenchymal cells during the enlargement phase, leading to rapid starch accumulation. Enzymes related to sugar metabolism and starch biosynthesis are upregulated during the enlargement phase, and the tuber also accumulates the storage protein dioscorin (Luo et al. 2018).

#### Phase III: maturation (October–November)

Phase III is characterized by photoperiod-dependent senescence of the leaf canopy, a decline in the accumulation of dry matter, and tuber maturation (Asiedu et al. 1998). Tuber cell size and starch accumulation reach maximum values during phase III (Luo et al. 2018). The end of tuber growth is accompanied by senescence of the stem and foliage, and the onset of complete tuber dormancy (Gong et al. 2017). Bulbils, if present, also reach their maximum size and enter dormancy at this point.

#### Phase IV: dormancy

For Chinese yam tubers and bulbils, the dormant phase lasts ~ 3 months and cold storage is required before dormancy is broken (Hashimoto et al. 1972; Okagami and Tanno 1991). The long dormancy phase allows cultivated yam tubers to be kept in prolonged storage. During storage, the tubers lose dry weight and moisture and undergo biochemical changes such as the loss of starch and the accumulation of the reducing sugars maltose and fructose (Hariprakash and Nambisan 1996). The characteristics of dormancy are well described in tropical edible yam species and the mechanisms have been extensively discussed elsewhere (Craufurd et al. 2001; Ile et al. 2006).

### Factors that influence tuber growth

The enlargement of yam tubers is influenced by the photoperiod. For example short-day (SD) conditions promote the enlargement of water yam tubers whereas long-day (LD) conditions have the opposite effect (Vaillant et al. 2005). Likewise, SD conditions (10-h days) promote tuber enlargement in Chinese yam, but only during the initial growth stage (Yoshida and Kanahama 1999; Chen et al. 2010). SD conditions inhibit tuber enlargement when the rapid growth phase is complete (Shiwachi et al. 2000). Furthermore, Chinese yam tuber growth is not limited to SD conditions, but continues (albeit more slowly) under LD conditions (Shiwachi et al. 2000). In the Chinese yam cultivar Nagaimo, SD conditions (8-h days) in the early to middle growth period produced larger new tubers (and bulbils) compared to LD (24-h light) conditions, but the tuber weight at harvest in December was the same in plants grown under LD and SD conditions (Yoshida et al. 2001; 2002).

Tuberization and tuber enlargement in Chinese yam seem to be controlled by plant hormones. For example, the endogenous concentration of biologically active gibberellins is related to tuber enlargement, increasing during tuber growth and reaching a peak at the beginning of the rapid growth phase (after 90 days). After that, gibberellin levels decline to low levels at the end of the growth phase (Gong et al. 2017). Accordingly, exogenous gibberellins can increase tuber yields (Yoshida et al. 2001, 2002; Kim et al. 2003b). However, gibberellins suppress bulbil formation, and the application of growth retardants such as mepiquat chloride (an inhibitor of gibberellin synthesis) can induce early bulbil formation and increase bulbil size in variety Tsukune (Yoshida et al. 2002; Kim et al. 2003a). These results indicate that underground tubers and aerial bulbils are regulated by different hormonal mechanisms. Breeding for larger and more abundant bulbils is important because the bulbils can only be used for propagation if they reach a certain size (Kim et al. 2003a).

The endogenous concentrations of other plant hormones also change during tuber growth: endogenous cytokinins and jasmonates accumulate during tuber enlargement and become depleted again during tuber maturation (Gong et al. 2017). Endogenous levels of the auxin indole-3-acetic acid (IAA) increase during tuber enlargement and reach a peak during the rapid growth stage. The concentration then declines during the late growth stage before increasing again, reaching a second peak during the maturation stage. The first peak may correlate with the induction of cell division by IAA during tuber enlargement, whereas the second may correlate with enhanced starch accumulation, although this has not been experimentally confirmed. Endogenous concentrations of abscisic acid (ABA) increase during the early stages of tuber enlargement and then decline continuously (Gong et al. 2017).

Plant hormones also influence tuber dormancy in Chinese yam (Hashimoto et al. 1972; Hasegawa and Hashimoto 1973). Batatasins are abundant in mature tubers but levels decline during prolonged storage (Kim et al. 2002). Exogenous gibberellins induce and extend dormancy in Chinese yam, suggesting that endogenous gibberellins participate in the induction and maintenance of dormancy (Okagami and Nagao 1971; Tanno et al. 1992; Okagami and Tanno 1993). Accordingly, sprouting is inhibited by the application of gibberellins and is promoted by gibberellin inhibitors (Okagami and Nagao 1971). The mechanisms behind the dual role of gibberellins in tuber enlargement and dormancy are not yet understood (Gong et al. 2017). Interestingly, gibberellins appear to have opposing functions in yam and potato tubers, given that gibberellins are required to break dormancy in potato (Hartmann et al. 2011; Rentzsch et al. 2012) but extend dormancy in cultivated yams (Okagami and Tanno 1993; Craufurd et al. 2001).

## Current research status and breeding objectives

China has domesticated several species of yam for food and medical purposes (Coursey 1967a; Wu et al. 2014) and there are 14 major cultivars of Chinese yam in Chinese market (Peng et al. 2017). Little is known about the genetics and genetic diversity of Chinese yam, although this knowledge would be invaluable for breeding approaches (Cao et al. 2018). Several molecular markers have been developed to study population diversity and to distinguish among cultivars, which is difficult to achieve using morphological characteristics alone (Mizuki et al. 2005b, 2010; Wu et al. 2014; Peng et al. 2017; Cao et al. 2018). Molecular markers are necessary to identify landraces and to prevent the misidentification of medical yam cultivars in China (Wu et al. 2014). Intron sequence amplified polymorphism (ISAP) and sequence characterized amplified region (SCAR) markers were therefore used to discriminate among the 14 major cultivars in China, leading to the identification of a marker specific for the most popular cultivar ‘Tiegun’, which is the sole source of tuber material used in traditional Chinese medicine (Peng et al. 2017). The authors detected high genetic diversity among these cultivars by ISAP profiling, which was explained by the domestication of yam driven by traditional farmers, who select for a broad range of traits. Favored varieties were therefore maintained and genetic erosion of the species was avoided (Peng et al. 2017). In the same study, ISAP markers were associated with morphological characteristics, including tuber and leaf shape (Peng et al. 2017). Recently, the chloroplast genomes of six Chinese yam cultivars were compared to develop new markers for genetic studies (Cao et al. 2018). Reference genome sequences have been published for *D. rotundata* and *D. dumetorum* (Tamiru et al. 2017; Siadjeu et al. 2020). A pre-publication genome sequence for *D. alata* is also available (Dioscorea alata v2.1 DOE-JGI, https://phytozome.jgi.doe.gov/) and preliminary analysis of the *D. zingiberensis* genome has been reported (Zhou et al. 2018) but was not yet made publicly available while this manuscript was in preparation. In addition, a bait kit to obtain genomic data for *Dioscorea* sp. has been designed improving the data situation for yam genotyping and phylogenetic comparison efforts (Soto Gomez et al. 2019). These assets indicate a growing interest in yam genetics although a genome sequence for *D. polystachya* is not yet available.

There have been few reports describing the molecular characterization of yam proteins *in planta* (Liu et al. 2017; Chen et al. 2019b). Genetic transformation has been successful in *D. rotundata* and *D. zingiberensis* (Zhu et al. 2009; Shi et al. 2012; Nyaboga et al. 2014) but the only transgenic yam line reported thus far is a *D. zingiberensis* mutant generated using the CRISPR/Cas9 system to reduce the expression of farnesyl pyrophosphate synthase mRNA, resulting in the depletion of squalene (Feng et al. 2018). The transformation of Chinese yam was recently confirmed, but the process is currently inefficient and must be optimized before routine applications are possible (Junhua et al. 2019). Micropropagation has been used to generate Chinese yam microtubers in tissue culture, which are useful to avoid the carryover of viruses during long-term vegetative propagation (Nagasawa and Finer 1989; Kohmura et al. 1995; Kadota and Niimi 2004; Li et al. 2014). Reference genes suitable for quantitative expression analysis have also been identified to enable further molecular studies (Zhao et al. 2016).

Several genes involved in storage organ development have been identified in potato and other tuberous crops (Navarro et al. 2011, 2015; Kloosterman et al. 2013; Lee et al. 2013). It is not yet clear if these genes also play a role in yam. Transcriptome analysis to compare tubers during initiation and expansion revealed several differentially expressed genes that may be involved in tuber expansion (Zhou et al. 2020). These included genes related to MAP kinase signaling, starch and sucrose metabolism, hormone biosynthesis and signaling, as well as transcription factors and miRNAs involved in these processes (Zhou et al. 2020).

Research is gathering pace in tropical yam species (Darkwa et al. 2019) but, as discussed above, breeding is hindered by the long growth cycle, dioecious reproduction, limited flowering, vegetative propagation, extensive polyploidy, and heterozygous genetic background (Mignouna et al. 2007). Crossing is also inefficient because fruiting is rare in female plants and the capsules often contain no seeds (Yakuwa et al. 1981). High temperatures (30 °C) have a positive effect on pollen germination and elongation, and hot summers could therefore promote seed setting in Chinese yam (Araki et al. 1981). Pollen from the cultivar Nagaimo can germinate and elongate in vitro, suggesting that impaired pollen germination cannot explain the lack of fruit and seed setting (Araki et al. 1981). Most research on Chinese yam currently takes place in East Asia, and many articles are published only in Chinese language journals without international access. These linguistic and technical barriers limit the global awareness of this crop and hamper research to improve its cultivation.

## Opportunities for the global cultivation of Chinese yam

Chinese yam was already used by humans in China during the Neolithic period: archaeological excavations at Xinglonggou confirm it was used as early as ~ 6200–5200 BC (Liu et al. 2015). There is further evidence from an archaeological site at Shuidonggou indicating its use as early as ~ 30,000 BC, during the Upper Paleolithic period (Guan et al. 2014). The species was fully domesticated ~ 1000 years ago (Peng et al. 2017) and was introduced into Europe as a potential alternative to potato during the Great Famine in Ireland caused by potato blight (Kühn 1855). Nowadays, Chinese yam is mostly unknown in Europe and is cultivated only in France near Orleans (O’Sullivan 2010) and in Germany near lake Constance. Comparatively high yields of 20 t/ha can be achieved in these regions based on FAOSTAT data from 2017 (Lebot 2019). More widespread cultivation is limited by the need for support during growth (Chinese yam is cultivated as an annual climber) and the lack of mechanical automated tuber-harvesting systems (Lebot 2019). Because the tubers of Chinese yam are delicate and become deformed when they encounter obstacles in the soil, it is important to identify candidate sites with optimal growth conditions. The Chinese yam is susceptible to various viruses including the *Japanese yam mosaic virus* (Fuji and Nakamae 1999; Mochizuki et al. 2017), *Yam mild mosaic virus* (Fuji et al. 2001), *Chinese yam necrotic mosaic virus* (Fukumoto and Tochihara 1978) and *Broad bean wilt virus 2* (Kondo et al. 2005). Leaf miners (*Acrolepiopsis* spp.) and root-knot nematodes have been reported to cause damage to Chinese yam cultivated in Japan (QianKui et al. 2000; Yasuda 2000; Tanaka et al. 2001). It can be assumed that related biotic stresses will also affect cultivation of Chinese yam in other regions of the world.

The most suitable areas in China are the flat landscapes in northern Shaanxi, eastern Shandong, and eastern Hebei, with specific soil parameters including a rich supply of minerals and a loose structure that allows the cultivation of fragile tubers (Fan et al. 2019). Although Chinese yam can be grown in humus or loess soil, sandy soil is preferable because it increases the polysaccharide and glucose content of the tubers (Ma et al. 2019). Warmer climates accelerate growth, and climate change may therefore make these areas even more suitable in the future, presumably reflecting the higher photosynthetic rate in response to elevated CO_2_ levels, leading to more leaf and tuber biomass under summer and autumn conditions, respectively (Thinh et al. 2017). The regions in China best suited for the cultivation of Chinese yam have also been predicted by spatial modeling using geographic information systems (Hu et al. 2018). This highlighted areas such as the lower reaches of the Yellow River basin and the North China Plain, mainly in north-eastern Henan, in the Hebei and Shandong provinces, largely in agreement with the results of soil analysis (Fan et al. 2019). In addition to the flat terrain and soil composed of loose, mineral-rich river sediments, these regions have an annual mean temperature of ~ 14 °C, an annual precipitation of 581 mm and ~ 2300 h of sunshine per year.

## Applications and innovations derived from Chinese yam

**Fig. 3:**
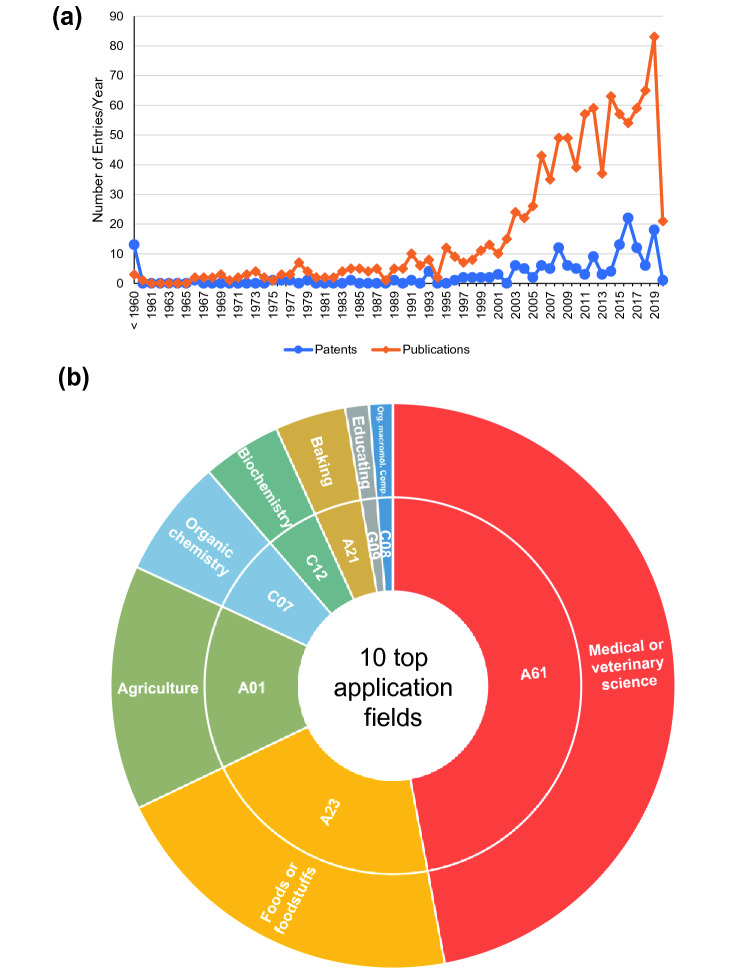
The increasing interest in Chinese yam over the last 20 years based on scientific publications and patents. **a** Annual number of scientific publications and patents referring to Chinese yam in title, abstract and/or keywords/claims (TS/CTB = (“Dioscorea polystachya” OR “shan yao” OR “shanyao” OR “iron yam” OR “Dioscorea batatas” OR “Dioscorea japonica” OR “Dioscorea decaisneana” OR “Dioscorea doryphora” OR “Dioscorea swinhoei” OR “Dioscorea rosthornii” OR “Dioscorea potaninii” OR “Dioscorea pseudobatatas” OR “Dioscorea opposita” OR “Dioscorea oppositifolia” OR “D. polystachya” OR “D. batatas” OR “D. japonica” OR “D. decaisneana” OR “D. doryphore” OR “D. swinhoei” OR “D. rosthornii” OR “D. potaninii” OR “D. pseudobatatas” OR “D. opposite” OR “D. oppositifolia” OR “Chinese yam” OR “cinnamon-vine” OR “Chinese potato” OR “nagaimo”); please note: given the confusion in the botanical nomenclature of the D*. polystachya*, our search term may include patents not necessarily referring to Chinese yam but other species as well. We have screened the patents; however we cannot guarantee that the true species accounted for is Chinese yam in every case) (publications from Web of Science since 1948; patents from Derwent World Patents Index (DWPI) since 1920). **b** Application fields based on international patent classification (IPC) codes, inner circle, for Chinese yam

As well as providing staple foods, yams are also used as functional foods and medicines. In this context, the rare ability of Chinese yam to grow in temperate regions is an advantage (Rinaldo 2020). China is currently the major exporter of Chinese yam products, followed by Mexico and the USA (https://www.tridge.com/intelligences/chinese-yam, retrieved on 02/11/2020). Europe holds a minor share of this market because Chinese yam products are also exported from France, but Europe as a whole is a major importer, with the UK, Netherlands, France and Germany all ranked among the top 10 importers. Globally, the top three importers are the USA, Japan and the UK. Patents mentioning Chinese yam reflect these market shares, with most published and registered in the US but with assignees in China or other parts of South East Asia (data not shown). The companies with the most patents/patent applications are L’Oreal (France), Standard Foods (Taiwan) and Sumitomo Chemicals (Japan). As discussed in more detail below, yam (*Dioscorea* sp.) tubers can also be used as a source of medicines or precursor compounds that are converted into drugs. For example, diosgenin and other steroids extracted from *D. villosa* or *D. composita* are used to synthesize pharmaceutically relevant derivatives such as cortisone (Lubbe and Verpoorte 2011). The importance of such applications is highlighted by Syntex Corporation (Panama), a company acquired by Roche in 1994 for more than US$ 5 billion (https://www.crunchbase.com/organization/syntex). The use of Chinese yam in traditional Chinese medicine was mentioned in the *Shennong Bencaojing* (The Classic of Herbal Medicine) first as a wild species, but almost exclusively in cultivated form for the last ~ 400 years (Cheng et al. 2014). The number of research publications and patents concerning Chinese yam has risen quickly over the last 20 years (**Fig. ****3**). Most of the patents refer to food or medical uses, but there are also patents describing harvesting machines (Aixin et al. 2004), mechanical seed dispensers (Ledermann and Stufflebeam 1998), agricultural chemicals (Hashimoto et al. 1979; Hayashi et al. 2009) and tissue culture techniques for in vitro propagation (Takayama and Akita 1991).

## Chinese yam ingredients with health benefits and other useful properties

Chinese yam tubers can be used directly as vegetables for cooking, but the starchy flour is also used for baking and for the preparation of noodles (Wang 2019; Nakagawa 2019; Li et al. 2020). The tubers not only provide calories and nutrients, but also contain various bioactive secondary metabolites that are likely to underlie the use of this species in traditional Chinese medicine and in modern herbal remedies (Perera and Li 2012). Chinese yam tubers and extracts have been shown to reduce serum levels of low-density lipoprotein (LDL) cholesterol and inhibit fat absorption in rodents, alleviating hypertension and attenuating obesity caused by a high-fat diet (Chen et al. 2003; Kwon et al. 2003; Liu et al. 2009; Amat et al. 2014; Gil et al. 2015). There is also evidence that the tubers reduce the incidence of diabetes (Gao et al. 2007; Hsu et al. 2007; Fan et al. 2015; Li et al. 2017), possibly reflecting their ability to inhibit α-glucosidase activity (Zhang et al. 2011). Chinese yam tubers are rich in antioxidants (Chiu et al. 2013) and have a positive effect on the immune system (Choi et al. 2004; Hou and Jin 2012) which is probably linked to their ability to modulate inflammation (Jin et al. 2011; Gil et al. 2015). Anti-tumor and prebiotic effects have also been reported (Huang et al. 2012; Liu et al. 2019; Zhang et al. 2019). Patents claiming pharmaceutical properties or health benefits have been approved for several Chinese yam extracts (Kotani et al. 2010; Nam et al. 2010; Kim et al. 2019) and powders (Wang et al. 2019). The ingredients with pharmacological activity are mainly polysaccharides (such as resistant starches), as well as sapogenins (especially diosgenin) and the storage protein dioscorin (Perera and Li 2012), which are discussed in turn below.

### Starches

Starch is the most abundant component of Chinese yam tubers and is the primary calorific constituent (as is the case for potato and cassava). Patents in this category include the use of Chinese yam starch to produce flours for instant noodles (Lometillo and Wolcott 1983) and animal feed such as horse biscuits (Naji and Nerys 1999). Other patents in the area of ‘medical and veterinary science’ indicate the potential utilization of Chinese yam starch as a functional food, including a drink ingredient that is purported to activate stem cell growth (Weng and Wu 2016).

Approximately 85% of the starch produced by Chinese yam plants is stored in the underground tubers for vegetative re-sprouting (Zhang et al. 2014, 2018), representing ~ 67% of the total dry weight of the tuber (Zhou et al. 2012; Zhang et al. 2018). Most of the rest is stored in the bulbils (Zhou et al. 2012; Zhang et al. 2018). The bulbil starch has greater swelling power, solubility in water and viscosity than tuber starch, and also greater thermostability and resistance to hydrolysis, reflecting the higher amylose content of 38% in bulbils compared to 35% in tubers (Zhang et al. 2018; Zhou et al. 2012). The amylose content is much higher than that found in other edible yam species, such as *D. rotundata* and *D. alata*, where the tubers contain 26% and 21% starch, respectively (Riley et al. 2004; 2014). The properties of Chinese yam starch are also cultivar dependent. For example, *D. opposita* cv. Baiyu starch has the lowest amylose content and the highest degree of crystallinity whereas *D. opposita* cv. Jiaxiangxichangmao starch has the highest amylose content and lowest degree of crystallinity (Shujun et al. 2006). As these results show, the degree of crystallinity is an indicator for the amylose content while the crystallinity type refers additionally to the amylopectin chain length (Cheetham and Tao 1998). Thus, high crystallinity (C_A_ and C_C_) are suggesting a higher amylopectin content however with a lower chain length and is positively influencing the resistance of starch against degrading enzymes (Cheetham and Tao 1998; Raigond et al. 2015). Both C_A_ (Zhang et al. 2018) and C_B_ (Shunjun et al. 2006) crystallinity patterns have been reported, and type C_C_ (a mixture of both) is likely to occur in Chinese yam tubers and bulbils (Shujun et al. 2008; Jiang et al. 2012). These results suggest a certain degree of amylopectin in Chinese yam tubers and bulbils. However only amylose content was directly measured yet, while a chemical determination of the starch composition is, to the best of our knowledge, still unavailable (Zhang et al. 2018; Chen et al. 2019a). The properties of Chinese yam starch could potentially be altered by the genetic modification of starch branching enzymes to favor amylose biosynthesis (Ma et al. 2018; Wang et al. 2018). Chinese yam starch shows higher gelatinization efficiency but a lower swelling potential compared to potato and maize starch (Shujun et al. 2008; Jiang et al. 2011). Chinese yam starch is stored mainly in amyloplasts, where (as discussed above) the starch granules influence gravitropism and thus tuber shape and growth (Kawasaki et al. 2001; 2008, 2014). Starch biosynthesis is therefore a valuable target for breeding strategies seeking to improve the nutritional, functional and/or developmental traits of Chinese yam.

The quality of extracted Chinese yam starch also varies with tuber freshness and drying method, affecting properties such as swelling power, solubility and gelatinization (Liu et al. 2019). These properties are important because they make the starch more resistant to digestive enzymes and support the growth of beneficial bacterial in the human microbiome. Accordingly, resistant starch can prevent hyperglycemic cycles and reduce the risk of diabetes (Birt et al. 2013; Raigond et al. 2015; Rinaldo 2020), and can improve colonic health and reduce the risk of colon cancer (Sajilata et al. 2006). Hot-air drying at 40 °C has the lowest impact on gelatinization, as well as preserving antioxidant activity and the content of polyphenols and allantoin, so this may be the ideal extraction method (Chen et al. 2017). Resistant starch makes up ~ 50% of the dry tuber weight (Nishimura et al. 2011; Jiang et al. 2012; Chen et al. 2017; Rinaldo 2020) and this may explain the ability of raw Chinese yam starch to lower the serum levels of triglycerides and LDL cholesterol in rodents (Shujun et al. 2008; Nishimura et al. 2011). The health benefits of starch are not only observed in mammals, but also in fish. Chinese yam peel, which is considered a waste product during the processing of tubers, improves the microbiome of the fish gut and can be used as an immunostimulatory feed additive (Meng et al. 2019b). Beyond food and medical applications, yam starch can also be used to create biofilms mixed with antimicrobial ingredients to protect food, such as pork meat and mayonnaise (Cheng et al. 2019a; Oyeyinka et al.^2017^), or in oxidized form in the paper manufacturing industry (Oyeyinka et al. 2017). These potential applications refer to yam starch in general and thus are applicable to Chinese yam.

### Other polysaccharides

The composition of Chinese yam mucilage polysaccharides is unclear, with contrasting studies reporting that they mainly comprise poly(β1-4)mannose with additional linkages and proteins (Ohtani and Murakami 1991), or a mixed composition of mannose, glucose, galactose and glucuronic acid (Ju et al. 2014). Polysaccharides can be extracted in hot water, followed by ethanol precipitation, without losing their functionality (Yang et al. 2015b). Extraction and purification can be challenging because the enzymatic treatment of mucilage polysaccharides to remove proteins also reduces their viscosity and alters other properties (Ma et al. 2018). Polysaccharides extracted from Chinese yam can improve insulin resistance and reduce cholesterol levels, limiting body weight in obese mice probably by inhibiting the uptake of saturated fatty acids (Cheng et al. 2019b). Similarly, anti-diabetic effects were observed in rats fed on diets supplemented with isolated Chinese yam polysaccharides (Fan et al. 2015; Yang et al. 2015a). Such polysaccharides also show antimicrobial activity (Yang et al. 2015c) and the ability to promote the growth of endometrial epithelial cells, suggesting they may help in the treatment of female sterility (Ju et al. 2014). However, this effect may be caused by diosgenin and its derivatives (see below), which are present at low levels in the polysaccharide extracts. Oligosaccharides generated by partial hydrolysis can be used as antioxidant food additives (Chen et al. 2015b). Similarly, low-molecular-weight mucilage extracts with a high content of uronic acid were shown to act as antioxidants with anti-mutagenic activity (Zhang et al. 2016). Seven patents have been granted based on Chinese yam polysaccharides and their derivatives, including a powdered formulation that promotes a healthy gut microbiome (Wang et al. 2019), a herbal medicine with hypoglycemic effects that also reduces the level of blood lipids (Wu 1997), and a mixture of polysaccharides and diosgenin formulated as a cream to facilitate wound healing, reduce skin inflammation and treat vein disorders (Eymard 2005).

### Diosgenin

Yams (*Dioscorea sp.*) synthesize multiple saponins and their aglycone derivatives (sapogenins), many of which show antifungal and cytotoxic effects (Sautour et al. 2007). Diosgenin is a steroidal sapogenin, similar in structure to cholesterol and the human hormones derived from it (Zagoya et al. 1971; Jesus et al. 2016). Diosgenin has therefore been identified as a drug candidate and is used as a precursor for the industrial synthesis of steroid hormones such as cortisone, pregnenolone, and progesterone (Edwards and Duke 2002; Chen et al. 2015a). Chinese yam produces both dioscin (a saponin) and diosgenin (its corresponding sapogenin) although the yield varies greatly in different reports and is likely to be dependent on the cultivar and growth conditions (Edwards and Duke 2002; Yang and Lin 2008; Yi et al. 2014; Wu et al. 2016). However, most of the diosgenin produced by the plant accumulates in the tuber flesh and cortex (Liu et al. 2010). Diosgenin is synthesized from squalene, which originates from the mevalonic acid pathway (Vaidya et al. 2013; Ciura et al. 2017; Hua et al. 2017; Zhu et al. 2018). Diosgenin can also be produced more homogeneously using *D. deltoidei* cell suspension cultures (Rokem et al. 1985) or genetically modified fungi, the latter also allowing the conversion of diosgenin into other sapogenins (Liu et al. 2010).

The functional significance of diosgenin and other steroidal sapogenins *in planta* has been comprehensively reviewed (Patel et al. 2012; Jesus et al. 2016; Chen et al. 2015a). Pharmacological applications have been claimed in at least 20 patents, describing drug compositions to reduce blood lipid levels (Shan et al. 2014) or diabetic neuropathy (Kim et al. 2014), as well as dermatological applications for the treatment of cellulite (Applezweig 1987) or wrinkles (Besne 2008). Diosgenin is also proposed as a treatment for cardiovascular disease, based on its ability to inhibit cholesterol uptake (Zagoya et al. 1971) and improve the lipid profile (Son et al. 2007) in rodent models. Prebiotic and anti-diabetic benefits also have been proposed because diosgenin can inhibit the activity of α-amylase and α-glucosidase (Huang et al. 2012; Arunrao Yadav et al. 2014). Interestingly, diosgenin has also been indicated for the treatment of Alzheimer’s disease and cancer (Patel et al. 2012; Chen et al. 2015a; Jesus et al. 2016). The former is based on clinical study data in which diosgenin-rich yam extract inhibited neurodegeneration and promoted cognitive stimulation (Tohda et al. 2017). The latter is based on the ability of diosgenin to induce apoptosis in tumor cells (Liu et al. 2005; Meng et al. 2019a) or trigger the corresponding signaling pathways (Raju and Mehta 2009). Finally, the antioxidant effects of diosgenin may reflect its ability to induce the expression and activity of enzymes such as catalase and superoxide dismutase, thus reducing the impact of reactive oxygen species (Son et al. 2007).

### Chinese yam storage proteins with functional properties – dioscorin and lectin

Although starch is the principal energy reserve in edible yam tubers, these organs also contain large quantities of storage protein (Shinjiro 1938). The protein content is interesting to the food industry, particularly the segment addressing the growing market for functional vegan foods and beverages (Wu 1998). The major soluble storage protein in Chinese yam tubers is dioscorin, which is a glycosylated form of the enzyme carbonic anhydrase. This means that the protein not only provides nutrition but also confers redox and antioxidant activity. In *D. alata*, dioscorin gene expression is dependent on meristematic activity during sprouting and tuberization, and is also regulated by environmental stimuli (Liu et al. 2017). Discorin remains stable and soluble following acidic extraction, and also retains its viscosity (Hu et al. 2018). It remains active over a broad pH range, conferring reducing and antioxidant activities presumably via disulfide–thiol exchanges (Hou et al. 1999, 2001; Shewry 2003; Chen et al. 2008; Xue et al. 2012). Despite the beneficial properties of dioscorin, we were unable to find any patents mentioning this protein specifically. Another major protein stored in Chinese yam tubers is a galactose-binding lectin. This is a defense protein that targets insect pests, suggesting it may be transferrable to other crops (Gaidamashvili et al. 2004; Ohizumi et al. 2009; Yoshimura et al. 2012). This protein has also been shown to inhibit cancer cell growth, and may therefore be useful as a lead to develop new cancer drugs (Yang et al. 2011; Chan and Ng 2013).

### Allantoin

Allantoin is major intermediate in the purine degradation pathway and accumulates in plants when uric acid is oxidized (Drewes and van Staden 1975). Allantoin can promote wound healing and cell regeneration, and is often included in skin lotions and other cosmetics (Fu et al. 2006). Yams (*Dioscorea sp*.) accumulate more allantoin than other tuberous crops, such as potato, sweet potato and cassava (Ozo et al. 1987). Chinese yam tubers contain 2–15 mg/g dry weight of this compound (Fu et al. 2006; Zhang et al. 2014; Liu et al. 2016; Wu et al. 2016) and it is more abundant in the skin than the flesh, making this part of the tuber highly valuable (Fu et al. 2006; Liu et al. 2016). The peel of Chinese yam tubers has greater antitumor activity than the flesh, which may reflect the higher content of allantoin, total phenolics and total flavonoids (Liu et al. 2016). Given that the peel is often discarded during processing, the utilization of this waste stream in a cascade-type approach would add value to the Chinese yam processing industry.

### Other potentially useful compounds

As well as the major components described above, other compounds present in smaller amounts may be useful in the context of functional foods and medical applications. For example, phenolic compounds from Chinese yam crude tuber extracts can protect against diabetes by promoting lipid degradation (Zhang et al. 2011; Yang et al. 2013). Specific effects have been attributed to particular phenolic compounds, such as the phenantrenes that accumulate in the peel (Kim et al. 2019). These confer anti-inflammatory, anticholinesterase, and antioxidant properties, as well as inhibiting the accumulation of triglycerides (Tóth et al. 2017). The batatasin family of phytohormones are classed as phenantrenes, and their ability to inhibit α-glucosidase may in part explain the anti-diabetic effects described above (Hu et al. 2015). Another potentially valuable ingredient is the DOI protein, which belongs to the chitinase-like superfamily but shows negligible chitinase enzyme activity (Wong et al. 2015). This protein can stimulate estradiol biosynthesis in rat cells, and is currently being investigated as a protein-based drug for the treatment of menopausal syndrome (Sze et al. 2013; Wong et al. 2015).

## The future of Chinese yam in Europe and beyond

Chinese yam has many properties that could help to address today’s challenges in the areas of food security, nutritional diversity, and diet-related health. More than 2 billion people worldwide are classed as obese (Ng et al. 2014), almost 18 million people die each year from obesity-related cardiovascular disease (Wang et al. 2014; Low Wang et al. 2016), and 415 million people suffer from diabetes (~ 90% type 2 diabetes, which is diet related), with the number predicted to exceed 600 million over the next decade (Jaacks et al. 2016). Diet-related health can be addressed using functional foods containing bioactive constituents with multiple health benefits, and this could be achieved by including additives based on Chinese yam extracts (Xiaoqun et al. 2012). Research focusing on yams (and Chinese yam in particular) has been largely overlooked in the past, at least in the West. This could be addressed by international collaborations that combine the traditional knowledge available in China with modern approaches in plant breeding, transformation, genome editing and metabolomics (Price 2017). Chinese yam could be integrated into a new circular bioeconomy, with the tubers and bulbils used to produce nutritional foods as well as pharmaceutical products, and the peel used for the extraction of bioactive compounds and as a functional feed for farm animals and the aquaculture industry.

### *Author contribution statement*

Both authors contributed equally to this work.
